# Protective effects of epigallocatechin-3-gallate counteracting the chronic hypobaric hypoxia-induced myocardial injury in plain-grown rats at high altitude

**DOI:** 10.1007/s12192-023-01386-1

**Published:** 2023-10-24

**Authors:** Haotian Chen, Chen Chen, Yuhui Qin, Lei Wang, Jie Zheng, Fabao Gao

**Affiliations:** 1 Department of Radiology, West China Hospital, Sichuan University, No. 37 Guoxue Road, Chengdu, 610041 China; 2 Mallinckrodt Institute of Radiology, Washington University School of Medicine, St. Louis, MO USA

**Keywords:** High altitude, Hypoxia, Myocardial injury, EGCG, Oxidative stress, Antioxidants

## Abstract

Exposure to hypobaric hypoxia (HH) environment causes stress to the body, especially the oxygen-consuming organs. Chronic HH conditions have adverse effects on the myocardium. Thus, we conducted this experiment and aim to evaluate such adverse effects and explore the therapeutic role of epigallocatechin-3-gallate (EGCG) in rats’ heart under chronic HH conditions. For that purpose, we transported rats from plain to a real HH environment at high altitude for establishing the HH model. At high altitude, animals were treated with EGCG while the salidroside was used as the positive control. General physiological data were collected, and routine blood test results were analyzed. Cardiac magnetic resonance (CMR) was examined to assess the structural and functional changes of the heart. Serum levels of cardiac enzymes and pro-inflammatory cytokines were examined. Oxidative markers in the left ventricle (LV) were detected. Additionally, ultrastructural and histopathological changes and apoptosis of the LV were assessed. Furthermore, the antioxidant stress-relevant proteins nuclear factor E2-related factor 2 (Nrf2) and the heme oxygenase-1 (HO-1) were detected. The experiment revealed that EGCG treatment decreased HH-induced elevation of cardiac enzymes and relieved mitochondrial damage of the LV. Notably, EGCG treatment significantly alleviated oxidative stress in the LV and inflammatory response in the blood. Western blot confirmed that EGCG significantly upregulated Nrf2 and HO-1. Therefore, EGCG may be considered a promising natural compound for treating the HH-induced myocardial injuries.

## Introduction

High altitude has a distinctive ecological environment characterized by hypobaric hypoxia (HH), low temperature, and intense ultraviolet solar radiation. This hostile environment has stress detrimental impact on the health of high-altitude residents and people who enter high plateau areas from low-altitude areas for either tourism or work (West 2017). Among the above adverse environmental factors, HH is considered the primary pathogenic one that causes hypoxemia and also tissue hypoxia, which may lead to acute or chronic mountain sickness (AMS or CMS) (Villafuerte et al. 2016; West 2017). The myocardial high oxygen consumption rate makes it sensitive to hypoxia. While a series of physiological adaptations of the cardiovascular system enable the organs to better cope with increased oxygen demand, the chronic HH conditions can still impair the myocardium (Jing et al. 2020; Chen et al. 2022). Therefore, understanding the biochemical mechanisms underlying chronic HH-induced myocardial impairment and exploring the therapeutic method has been a focus of research in high-altitude medicine.

Recent researches have revealed that the cardiovascular system can be adversely affected by the HH-induced oxidative stress (Pena et al. 2020; He et al. 2021). Generally, the reactive oxygen species (ROS) are considered the physiological regulator reflecting the cellular redox status. However, at high altitudes, as excessively produced ROS cannot be effectively scavenged by endogenous antioxidant enzymes, oxidative stress is generated and consequently causes detrimental effects to the cells, leading to pathophysiological conditions (Gaur et al. 2021). Additionally, chronic HH conditions may be associated with inflammatory response, which further aggravate myocardial dysfunction (Wang et al. 2016; Pham et al. 2021). Accordingly, strategies that against oxidative stress and inflammatory response were proposed for treating HH-induced myocardial impairment (Dosek et al. 2007; Gaur et al. 2021).

It is against this backdrop that natural compounds have been put forward as a novel option for the treatment of cardiovascular diseases owing to its broad spectrum of efficacy and few side effects compared with synthetic compounds (Eng et al. 2018). Tea is popular worldwide due to its distinct flavor and potential benefits to health. Epigallocatechin-3-gallate (EGCG), which makes up almost 40% of the green tea polyphenols, is well known for its antioxidant activity (Eng et al. 2018). Recent researches have shown that EGCG possesses pharmacological properties for treating various diseases, including cardiovascular diseases, neurodegenerative diseases, diabetes, and cancers, ranging from preclinical to clinical studies (Talebi et al. 2021; Hu et al. 2022). It has been reported that EGCG can directly neutralize free radicals and activate antioxidant enzymes to indirectly exert antioxidant effects. Additionally, EGCG supplementation can also scavenge inflammatory cytokines. Nevertheless, to date, the efficacy of EGCG for HH-induced myocardial impairment remains to be evaluated. Also, its potential cardioprotective role at high altitudes remains to be investigated. In this experiment, we used another herb, salidroside, as a positive control. As the primary active component of *Rhodiola rosea* *L*., salidroside is utilized for preventing and treating HH-associated diseases in many countries (Han et al. 2022). Its cardioprotective role on HH-induced myocardial impairment has been reported (Hsu et al. 2017).

Given that the animal models are of great value to explore the pathophysiological outcomes of the hypoxia-related human diseases (Barnes et al. 2022), in this experiment, the HH rat model was established in a genuine high-altitude environment instead of a simulated one. Based on the rat model, our aim was to investigate the detrimental effects of high-altitude HH environment on the myocardium, focusing on the perspectives of pathology and biochemistry. Furthermore, the potential cardioprotective mechanism of EGCG was explored.

## Materials and methods

### Study protocol

**Fig. 1:**
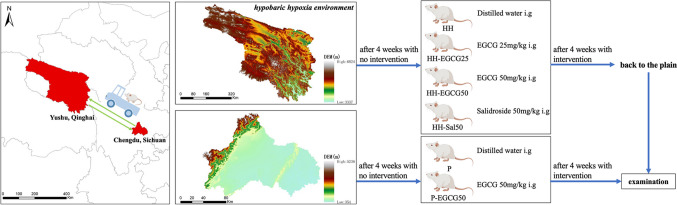
Experimental protocol (parts of the elements provided by Figdraw (www.figdraw.com))

Seventy-two male Sprague–Dawley rats (130–150 g), provided by Chengdu Dossy Experimental Animals Co., Ltd. (Dossy, Chengdu, China), were randomized into the high-altitude group as experimental group (*n*=48) and plain control group (*n*=24). Those rats were allowed free access to standard maintenance diet and water under well-controlled environment. They were fed in separate cages (5 in each cage). Animals in high-altitude group were transported from Chengdu, China (an altitude of 500 m) to Yushu, China (an altitude of 4250 m). Four weeks after the arrival, rats were averagely allocated into another 4 groups, viz., HH group, EGCG 25 mg/kg (HH-EGCG25 group), EGCG 50 mg/kg (HH-EGCG50 group), and salidroside 50 mg/kg (HH-Sal50 group). EGCG (Sigma-Aldrich, Inc.) and salidroside (Sigma-Aldrich, Inc.) were dissolved in distilled water for 30 min before treatment and given by gavage every day for 4 weeks. Meanwhile, 24 rats from the plain control group, fed and housed in Chengdu, China, were averagely allocated into 2 groups, viz., control (P group) and EGCG 50 mg/kg (P-EGCG50 group). After the first 4 weeks with no intervention, the P-EGCG50 group received medication by gavage every day for another 4 weeks. In the meantime, rats in the P group and HH group were gavage by the same volume of distilled water as described in Fig. 1. The gavage of EGCG, salidroside, and distilled water was given to the rats every morning after an overnight fast (12 h). The dose of EGCG and salidroside used was selected according to the methods in previous articles (Hsu et al. 2017; Eng et al. 2018).

### Blood sample analysis

After 8 weeks of stay in the HH environment, the rats in the experimental group were transported back to Chengdu, China. After the arrival, we immediately collected the blood samples. The whole blood (1 mL) samples used for routine blood tests were determined by automated cell analyzer (Mindray, BC-11, Shenzhen, China). The serum samples were obtained by coagulating the whole blood (2 mL) for 20 min and centrifuging at 4000 rpm for 15 min. Serum levels of cardiac biomarkers including creatine kinase (CK), creatine kinase MB isoenzyme (CK-MB), and lactic dehydrogenase (LDH) were measured by automatically validated methods and equipment (Mindray, BS360S, Shenzhen, China). Serum levels of erythropoietin (EPO), cardiac troponin I (cTnI), and inflammatory cytokines including tumor necrosis factor-α (TNF-α), interleukin-1β (IL-1β), and IL-6 were tested through enzyme-linked immunosorbent assay (ELISA) kits (Elabscience, Wuhan, China).

### CMR protocol and analysis

The cardiac alternations of structure and function were noninvasively assessed using CMR. The CMR scan was performed in an ultra-high-field 7T dedicated device (Bruker, Ettlingen, Germany), and its protocol was used as the same of our previous report (Zhang et al. 2022). The animals were placed prone on the scanning bed under anesthetized condition. The electrocardiography and the respiration were monitored. A heating blanket was placed under the abdomen of the rats to keep it warm. After the localization images were scanned, a standard fast low angle shot (FLASH) sequence was performed to acquire the dynamic images of the cardiac cycle. A total scanning time was about 50 min. After that, we manually draw the epicardium and endocardium of the LV by using SD short module of the CVI42 software (Calgary, Alberta, Canada). The LV structural and functional parameters including left ventricular end-diastolic volume (LVEDV), end-systolic volume (LVESV), stroke volume (LVSV), ejection fraction (LVEF), and left ventricular mass were obtained. The cardiac output was calculated by multiplying stroke volume (SV) by heart rate. Furthermore, the cardiac index (CI) was defined as the cardiac output divided by the body weight.

### Transmission electron microscopy (TEM)

The hearts were collected and washed after the CMR scan. Myocardial tissues (≤ 1 mm^3^) were quickly and carefully acquired from the LV in the cold fixative and were fixed in glutaraldehyde at 4 °C for 24 h. Following that, the specimens were dehydrated in ethanol at room temperature and embedded into the pure EMBed 812 at 37 °C overnight. The samples were cut and stained with uranyl acetate and lead citrate. The images were observed and obtained under TEM. Subsequently, the Flameng method was utilized to evaluate and score the degree of mitochondrial damage in each group of rats (Flameng et al. 1980). We randomly chose five fields from each rat and selected 20 mitochondria from each field in a randomized manner for scoring (100 mitochondria per rat). The total scores of 100 mitochondria per rat were calculated by summing them and then dividing the sum by 100. This ratio represents the degree of mitochondrial damage, with higher ratios indicating more severe injury.

### Oxidative stress assessment

The LV tissues were homogenized and centrifuged (8000 rpm, 15 min, 4 °C). Following that, the supernatants were collected for oxidative stress tests. The malondialdehyde (MDA), superoxide dismutase (SOD), and glutathione peroxidase (GSH-Px) were assessed matched to the supplier’s instructions (Elabscience, Wuhan, China). The intact cardiac tissues stored at −80 °C were used to make frozen preparations. 2,7-Dichlorofluorescein diacetate (DCFH-DA) was applied for observing the cytosolic superoxide anion to assess the reactive oxygen species (ROS) production in the myocardium. To quantify the ROS level, DCFH fluorescence intensity was measured using a confocal microscope (Nikon, A1RMP+, Japan).

### Histopathological and TUNEL assay

Forty-eight hours after fixation in 4% paraformaldehyde, the myocardial specimens were dehydrated, paraffinized, and sliced along the short axis (5 mm thickness). The degree of myocardial deposition of collagen fibers was assessed by Masson’s trichrome (MT) staining. In addition, the hematoxylin-red complex picric acid (HBFP) staining was used for identifying the extent and region of the hypoxic myocardium. Pathological images were observed and captured under the optical microscope (Leica, DM500, Germany). The histopathological changes such as collagen volume fraction (CVF) were quantitatively analyzed by the ImageJ software (National Institutes of Health, USA).

Myocardial apoptosis was detected by terminal deoxynucleotidyl transferase-mediated dUTP nick-end labeling (TUNEL) and 4′,6-diamidino-2-phenylindole (DAPI) staining. We collected images through fluorescence microscope (DAPI emits blue light and FITC emits green). The percentage of myocardial apoptosis was calculated by dividing the number of TUNEL-positive cells by the total number of cardiac cells counted.

### Western blot

The procedure and protocol for Western blot analysis followed that of our recent report (Chen et al. 2022). In the same way, proteins of the cardiac tissues were lysed by the RIPA lysis buffer, and the protein concentrations were measured with the bicinchoninic acid assay kit (BCA, Servicebio, Wuhan, China). Protein samples were electrophoresed on SDS-PAGE and transferred to the polyvinylidene fluoride (PVDF) membranes (Servicebio, Wuhan, China), which were blocked with 5% nonfat milk for 2 h. The membranes were subsequently incubated with the following primary antibodies against Nrf2 (1:2000, ImmunoWay, TX, USA), Bax (1:1000, Servicebio, Wuhan, China), HO-1 (1:1000, Affinity Biosciences, Jiangsu, China), Bcl-2 (1:1000, Ibid.), caspase-3 (1:1000, Ibid.), and β-actin (1:5000, Ibid.) at 4 °C overnight. Subsequently, the membranes were then incubated with horseradish peroxidase (HRP)-labeled secondary antibody solution at room temperature for 1 h. The proteins were visualized by enhanced chemiluminescent (ECL, Biosharp, Hefei, China) and detected by ChemiScope 6100 imaging system (Clinx, Shanghai, China).

### Statistical methods

The experimental data were represented as mean ± standard deviation (SD). The GraphPad Prism version 9.5.0 (GraphPad Software, San Diego, CA, USA) was used for statistical analysis. One-way variance analysis was employed to calculate the differences followed by Tukey’s multiple comparison test. *P* < 0.05 was considered statistically significant.

## Results

### HH environment affects the general physiological data

No mortality was observed in all 6 groups throughout the experiment. The body weight, respiration rate, and heart rate of the rats across the groups showed no significant difference (Table 1). Several main hematological parameters reflecting the capacity of O_2_ delivery are listed in Table 1. The red blood cell (RBC), hemoglobin (HGB), hematocrit concentration (HCT), mean corpuscular hemoglobin concentration (MCHC), and erythropoietin (EPO) levels of the HH group increased significantly in comparison with those of the P group, indicating that the HH rat model was successfully established. Additionally, using EGCG and salidroside did not reduce such compensatory effects of the hematopoietic system under the HH environment. At the same time, the levels of white blood cell (WBC) and platelet (PLT) remained within normal limits in HH group.

**Table 1:** Comparison of general physiological data for rats among six groups

**Index**	**P**	**P-EGCG50**	**HH**	**HH-EGCG25**	**HH-EGCG50**	**HH-Sal**
Body weight (g)	387.83±14.74	380.83±17.06	368.00±20.39	364.40±16.86	368.00±17.31	374.20±43.03
Respiration (times/min)	40±4	38±2	40±2	40±3	40±2	39±3
Heart rate (beats/min)	290±15	301±12	298±8	298±11	295±14	294±15
RBC (10^12^/L)	8.35±0.28	8.57±0.51	9.02±1.04^*^	9.30±0.40	9.02±0.58	9.16±0.46
HGB (g/L)	159.63±3.50	159.88±7.59	182.20±19.24^***^	188.20±9.98	181.50±8.35	185.00±11.49
MCHC (g/L)	356.38±2.00	357.50±4.07	374.40±5.41^***^	360.20±9.78	368.00±2.16	375.00±6.58
HCT (%)	44.84±1.02	44.70±1.78	48.68±5.59^*^	52.22±1.74	49.40±2.40	49.30±3.84
EPO (pg/mL)	227.05±51.05	221.24±81.31	498.82±86.41^***^	480.23±126.19	482.65±143.36	461.59±105.61

*RBC*, red blood cell; *HGB*, hemoglobin; *MCHC*, mean corpuscular hemoglobin concentration; *HCT*, hematocrit; *EPO*, erythropoietin. ^*^P < 0.05 and ^***^P < 0.001 compared with the P group. Values are expressed as mean ± SD

### HH environment affects the structure and function of the LV

CMR scan was performed to evaluate the cardiac structure and function because it can provide excellent resolution and repeatability of soft tissue. LV parameters are summarized (Table 2). Compared with the P group, LVEDV, LVESV, and LV mass in rats of the HH group showed a significant decrease, while the LVSV, LVEF, and CI were preserved. These alternations were not affected by EGCG and salidroside treatment.

**Table 2:** The structural and functional parameters in the LV acquired from CMR among six groups

**Index**	**P**	**P-EGCG50**	**HH**	**HH-EGCG25**	**HH-EGCG50**	**HH-Sal**
LVEDV (mL)	0.46±0.02	0.45±0.02	0.41±0.04^*^	0.44±0.03	0.42±0.05	0.42±0.05
LVESV (mL)	0.15±0.01	0.14±0.01	0.12±0.01^*^	0.13±0.02	0.14±0.02	0.13±0.01
LVSV (mL)	0.32±0.01	0.31±0.01	0.28±0.04	0.31±0.01	0.28±0.05	0.29±0.05
LVEF (%)	69.54±1.01	68.99±1.13	67.39±2.13	68.24±1.75	68.33±2.71	69.35±2.54
LV mass (g)	0.42±0.04	0.40±0.06	0.36±0.04^*^	0.38±0.02	0.39±0.05	0.40±0.03
CI (mL/min/g)	0.24±0.01	0.23±0.02	0.23±0.04	0.26±0.02	0.23±0.04	0.23±0.02

*LVEDV*, left ventricular end-diastolic volume; *LVESV*, left ventricular end-systolic volume; *LVSV*, left ventricular stroke volume; *LVEF*, left ventricular ejection fraction; *CI*, cardiac index. ^*^P < 0.05 compared with the P group. Values are expressed as mean ± SD

### EGCG reduces the HH-induced injuries of the LV

**Fig. 2:**
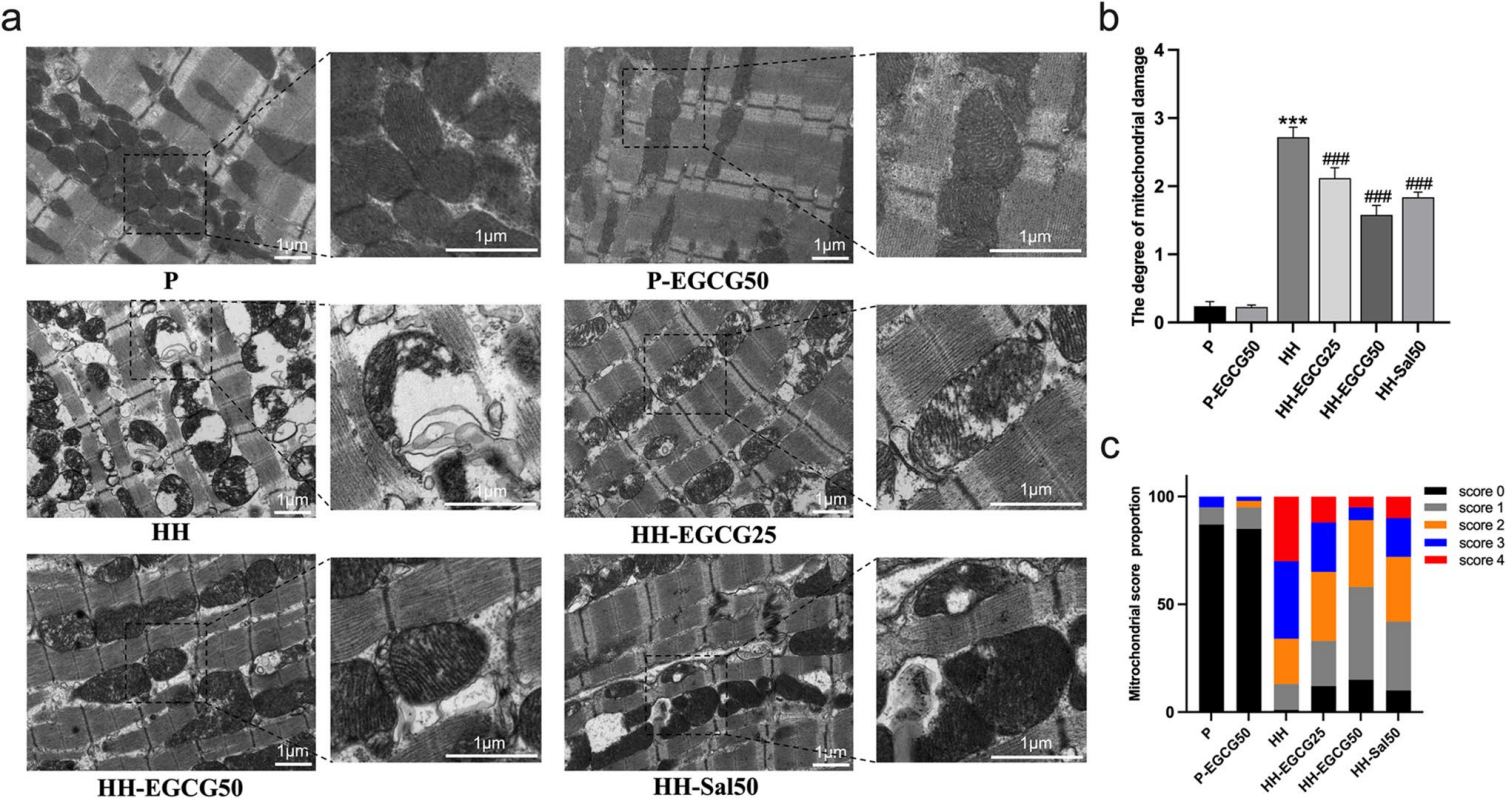
Transmission electron microscopy of the myocardium demonstrates that the mitochondria were severely affected by the chronic HH exposure. **a** Representative TEM images of the P, P-EGCG50, HH, HH-EGCG25, HH-EGCG50, and HH-Sal50 groups. **b**, **c** Semi-quantitative analysis of mitochondrial damage in each group. ***P < 0.001 compared with the P group; ###*P* < 0.001 compared with the HH group

As shown in Fig. 2a, transmission electron microscopy (TEM) demonstrates that the ultrastructure and shape of the myocardium from both the P and P-EGCG50 groups were normal. The myocardial fibers were orderly arranged, and the mitochondria appeared well-integrated structure with densely packed cristae. However, after 8 weeks of chronic HH exposure, a large number of myocardial mitochondria from the HH group became swollen, and the mitochondrial crests were decreased and disordered in shape, with some of them vacuolated and broken. In contrast, EGCG and salidroside treatment significantly reduced the HH-induced mitochondrial injuries, with the HH-EGCG50 group excelling in performance. By using the Flameng method, the degree of mitochondrial damage of LV among groups was semi-quantitatively analyzed and demonstrated in Fig. 2b and c. The score in the HH group was significantly higher than the P group and was significantly reduced following treatment with EGCG and salidroside.

**Fig. 3:**
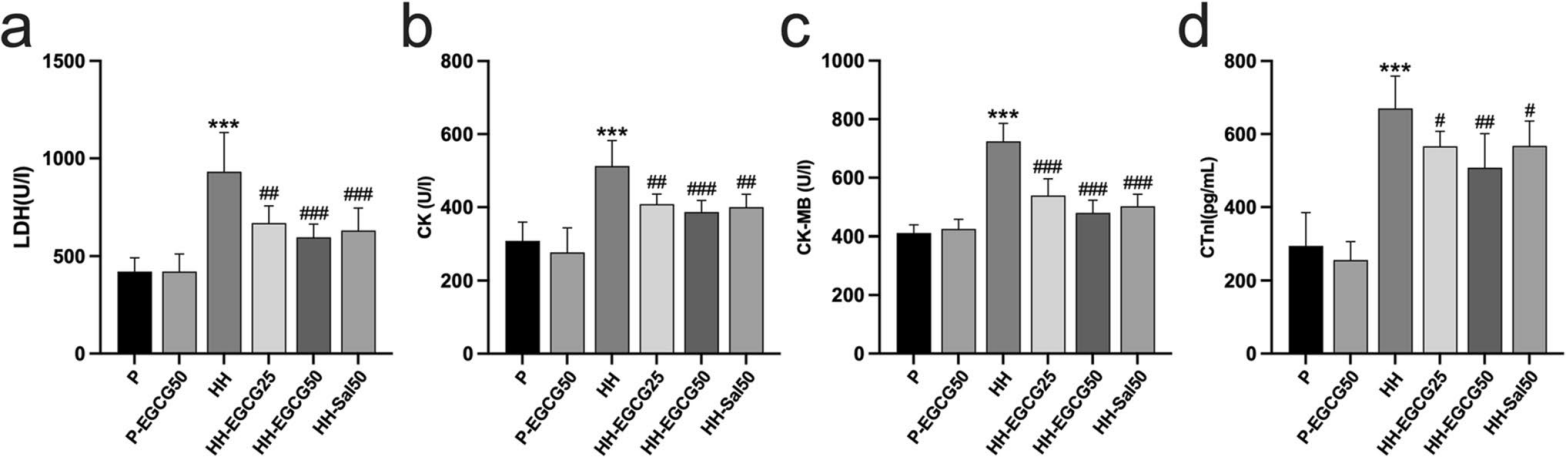
Serum levels of cardiac biomarkers include **a** LDH, **b** CK, **c** CK-MB, and **d** CTnI in different groups. ***P < 0.001 compared with the P group; #*P* < 0.05, ##*P* < 0.01, and ###*P* < 0.001 compared with the HH group

Serum levels of cardiac biomarkers including LDH, CK, CK-MB, and cTnI (Fig. 3a–d) were then measured. Statistical analysis of these biomarkers between the HH group and P group demonstrated significant differences, suggesting that an 8-week HH exposure had detrimental effects on the rat heart. Compared with the HH group, the augment of these biomarkers in HH-EGCG25, HH-EGCG50, and HH-Sal50 groups was significantly suppressed.

### EGCG ameliorates the HH-induced oxidative stress and inflammatory responses

**Fig. 4:**
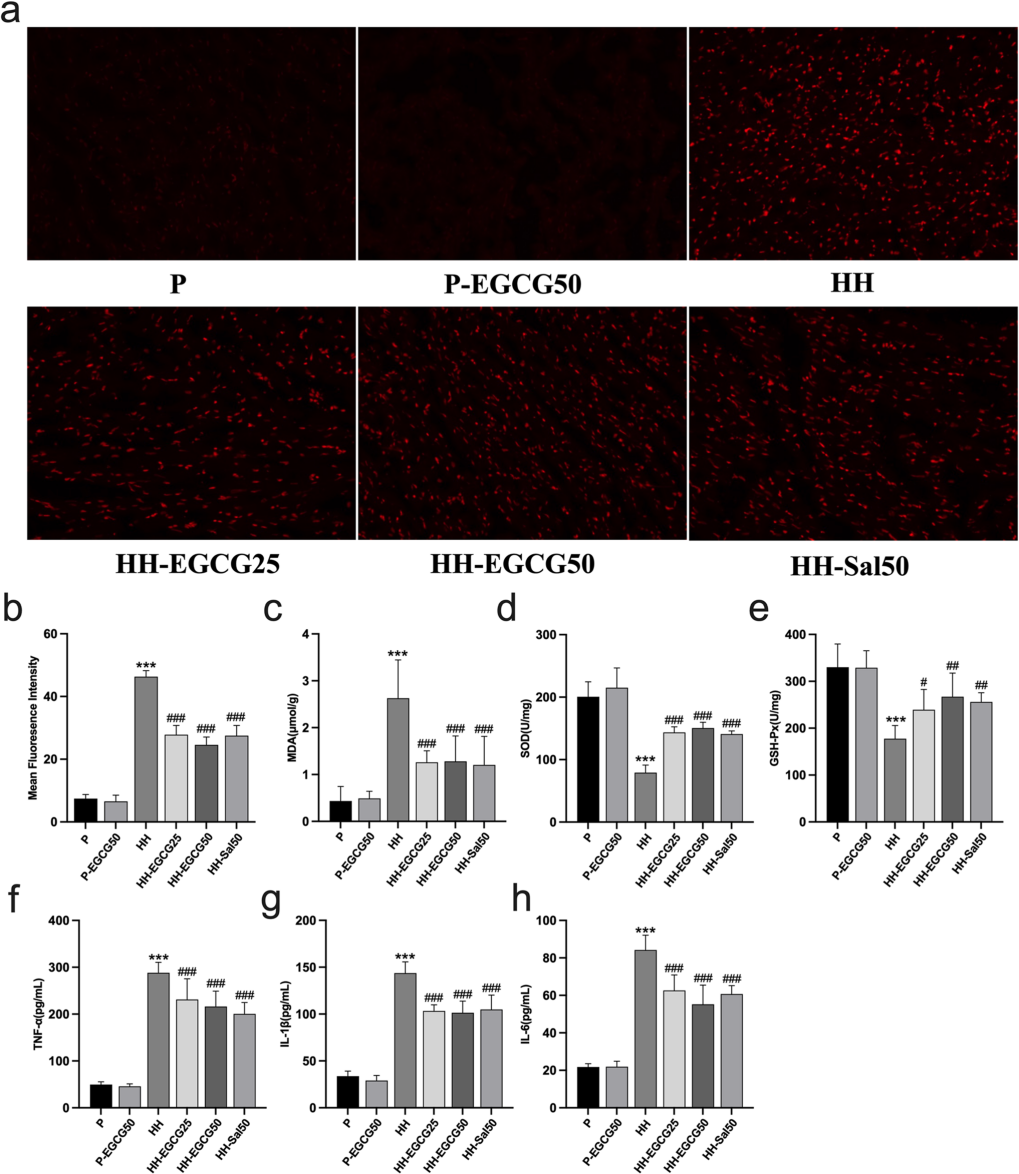
EGCG and salidroside ameliorated HH-induced oxidative stress and inflammatory responses. **a** ROS generation detected by DCFH-DA staining of the P, P-EGCG50, HH, HH-EGCG25, HH-EGCG50, and HH-Sal50 groups. **b** The values of fluorescence intensity which stand for level of ROS generation. **c**–**e** The concentration of MDA, SOD, and GSH-Px in the cardiac tissues, respectively. **f**–**h** Serum levels of pro-inflammatory cytokines including TNF-α, IL-1β, and IL-6, respectively. ***P < 0.001 compared with the P group; #*P* < 0.05, ##*P* < 0.01, and ###*P* < 0.001 compared with the HH group

We detected oxidative stress-related biomarkers in the LV tissues. The representative images of ROS staining of frozen sectioned LV in each group are shown in Fig. 4a. The values of fluorescence intensity, which stands for the levels of ROS generation, are shown in Fig. 4b. Fig. 4c shows the levels of MDA, the index of lipid peroxidation, which reflects the ROS damage. According to the results, the LV of the HH group exhibited a significant increase in ROS production and MDA concentration compared with the P group. However, EGCG and salidroside treatment effectively reversed these effects. We subsequently explore the influence of EGCG on the antioxidant enzymes including SOD and GSH-Px in the LV (Fig. 4d, e). Compared with the P group, exposure to chronic HH conditions led to a significant reduction in the activities of SOD and GSH-Px. However, EGCG-treated rats showed higher activities of these enzymes than the HH group with statistical significance.

We also examined effects of EGCG on inflammatory responses induced by HH exposure (Fig. 4f–h). ELISA tests demonstrated a significant increase in serum levels of TNF-α, IL-1β, and IL-6 in the HH group compared with the P group. However, compared with the HH group, the increase in pro-inflammatory cytokines was significantly reduced by using EGCG. Similar results were obtained in the HH-Sal50 group. These findings reveal that EGCG may exert a cardioprotective role by ameliorating HH-induced oxidative stress and inflammatory responses.

### EGCH reduces HH-induced collagen deposition and anoxic area of the LV

**Fig. 5:**
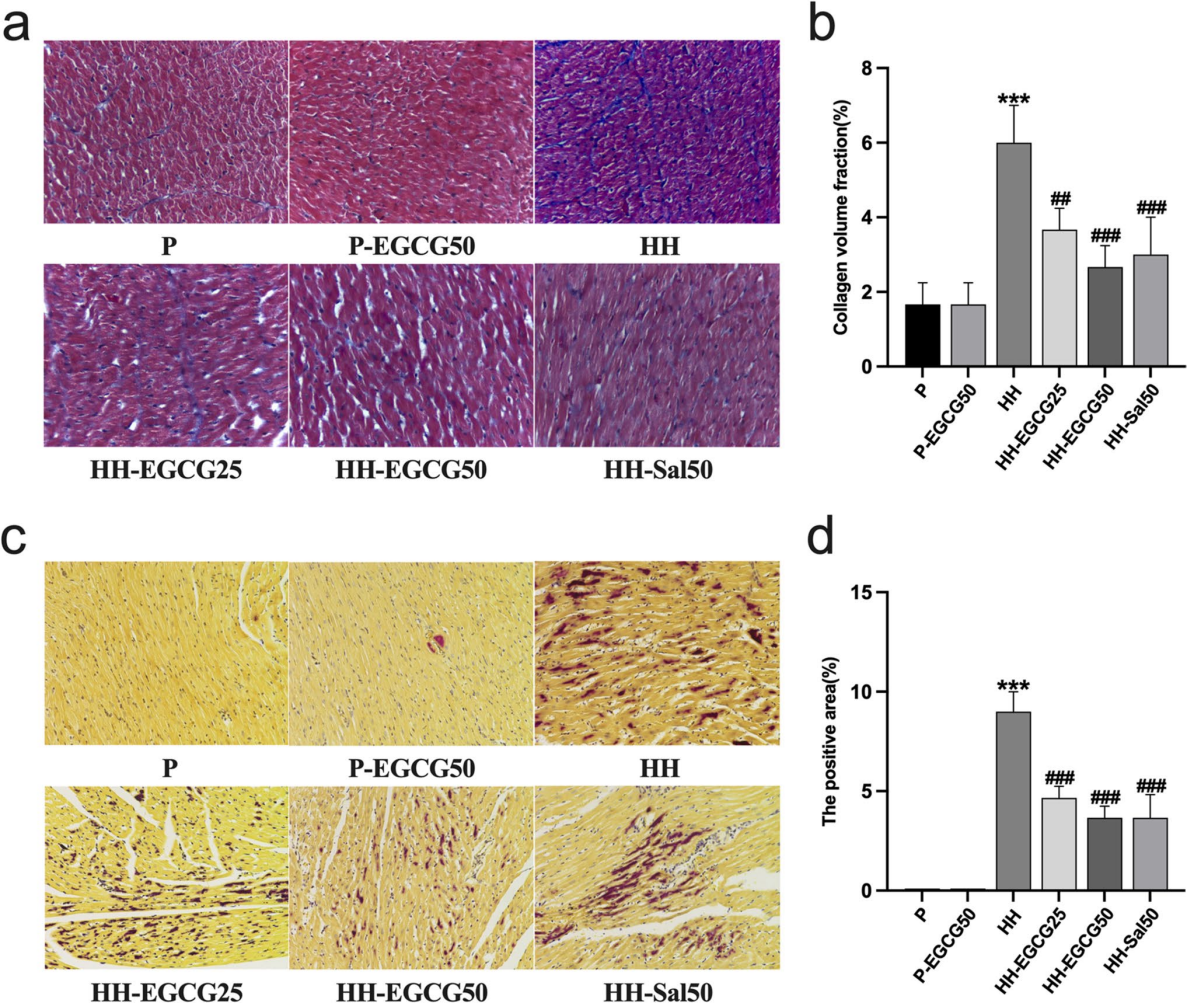
EGCG and salidroside reduced HH-induced myocardial fibrosis and myocardial hypoxia. **a** Representative photomicrographs of the P, P-EGCG50, HH, HH-EGCG25, HH-EGCG50, and HH-Sal50 groups stained with Masson’s trichrome (× 400). **b** Quantification of collagen volume fraction in the cardiac tissues. **c** Representative HBFP staining of the P, P-EGCG50, HH, HH-EGCG25, HH-EGCG50, and HH-Sal50 groups (× 200). **d** Quantification of the anoxic area in the cardiac tissues. The results are expressed as mean ± standard deviation (SD). ***P < 0.001 compared with the P group; ##*P* < 0.01 and ###*P* < 0.001 compared with the HH group

Representative photomicrographs of cardiac tissue sections stained with Masson’s trichrome are shown in Fig. 5a. The connective tissue was stained blue, the nuclei was purple, and the cytoplasm was red/pink. It was shown that the CVF of the LV was significantly higher in the HH group than in the P group, and the EGCG and salidroside treatment alleviated this effect (Fig. 5b). Statistical analysis of the CVF showed no significant difference among the HH-EGCG25, HH-EGCG50, and HH-Sal50 groups. On HBFP staining, the myocardial tissue stained yellow/brown was normal while the scarlet tissue was anoxic. As shown in Fig. 5c, a large number of cardiomyocytes in the HH group were stained scarlet. As expected, the anoxic area significantly reduced in the HH-EGCG25, HH-EGCG50, and HH-Sal50 groups compared with the HH group (Fig. 5d).

### EGCG suppresses the HH-induced apoptosis of the LV

**Fig. 6:**
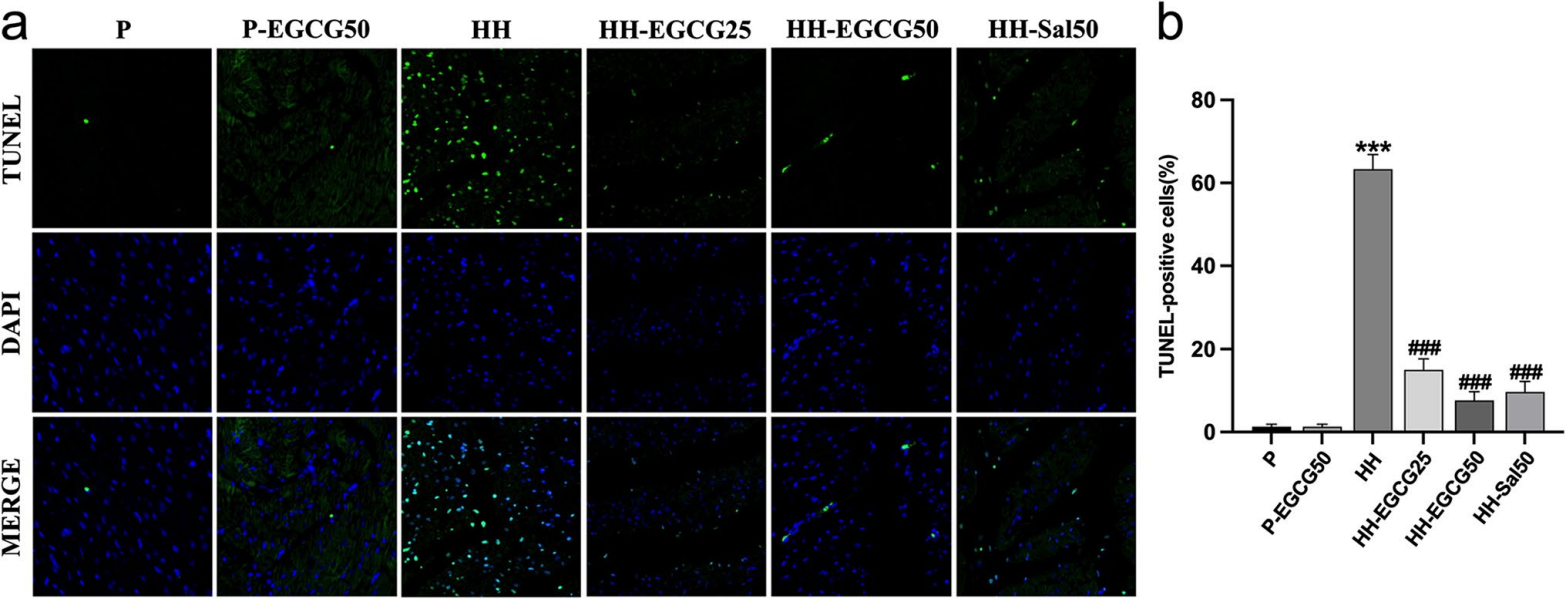
EGCG and salidroside suppressed HH-induced myocardial apoptosis, determined by the TUNEL assay. **a** Apoptotic cells stained green, and nuclei stained blue with DAPI. **b** Quantitative apoptosis analysis. ***P < 0.001 compared with the P group; ###*P* < 0.001 compared with the HH group

**Fig. 7:**
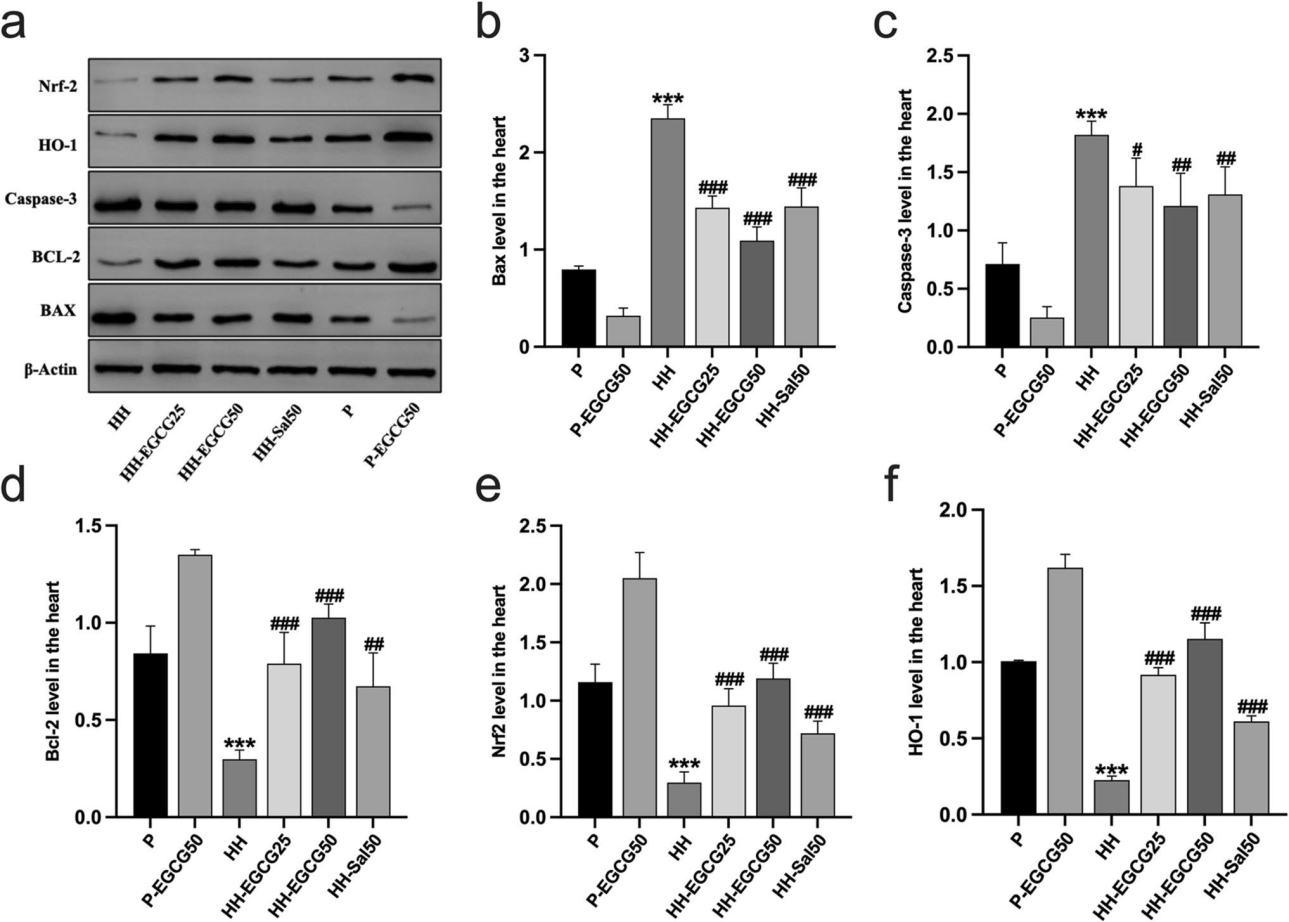
EGCG and salidroside suppressed HH-induced myocardial apoptosis and regulated the expression of antioxidant-related proteins Nrf2 and HO-1. **a** Protein levels of Nrf2, HO-1, Bax, caspase-3, and Bcl-2 in the LV tissues were measured by immunoblotting. The band intensity of **b** Bax, **c** caspase-3, **d** Bcl-2, **e** Nrf2, and **f** HO-1 was quantified. Nrf2, nuclear factor E2-related factor 2; HO-1, heme oxygenase-1; LV, left ventricle. **P < 0.01 and ***P < 0.001 compared with the P group; #*P* < 0.05, ##*P* < 0.01, and ###*P* < 0.001 compared with the HH group

The myocardial apoptosis was determined by TUNEL staining (Fig. 6). The normal cardiomyocyte nuclei were stained blue while the apoptotic one were stained green (Fig. 6a). The data analysis showed a significantly higher apoptosis rate of cardiomyocytes in the HH group compared with the P group. However, EGCG and salidroside significantly decreased the number of apoptotic cardiomyocytes compared with the HH group (Fig. 6b). Furthermore, we examined the apoptosis-related protein expression in the LV tissues, including Bax, caspase-3, and Bcl-2 (Fig. 7a). The results indicated a significant increase in pro-apoptotic protein levels of Bax (Fig. 7b) and caspase-3 (Fig. 7c) and a decrease in anti-apoptotic protein Bcl-2 (Fig. 7d) in the HH group compared with the P group. However, EGCG treatment remarkably reversed these abnormal trends of protein expression and protected the myocardium from HH-induce apoptosis. Similar results were observed in HH-Sal50 group.

### EGCG upregulates the Nrf2 and HO-1 in the LV

To gain a deeper understanding of the cardioprotective mechanism of EGCG in rats exposed to chronic HH conditions, we analyzed total expression status of Nrf2 and HO-1 in the LV (Fig. 7a). HO-1, a phase II detoxification enzyme regulated by Nrf2, exerts a crucial role in cellular protection. Western blot showed that protein expression of Nrf2 and HO-1 (Fig. 7e, f) remarkably decreased in the HH group after exposure to chronic HH conditions compared with the P group. EGCG and salidroside treatment significantly upregulated Nrf2 and HO-1 expression in HH-EGCG25, HH-EGCG50, and HH-Sal50 groups compared with the HH group.

## Discussion

At high altitude, HH is the main factor that cause people to feel unwell and can cause hypoxemia and tissue hypoxia (West 2017). Under HH conditions, cellular oxidative stress is enhanced and causes potential damage to the cardiomyocytes. Therefore, anti-oxidative supplementation may be a viable solution to address this problem. EGCG was reported to exert positive role on cardiovascular disorders with no significant adverse effects at a therapeutic dose (Clement 2009; Ferenczyova et al. 2021). Previous researches have shown that EGCG benefits hypoxia-induced myocardial impairment in models. For instance, EGCG was found to improve the cardiomyocyte viability after hypoxia/reoxygenation (H/R) injury while alleviating mitochondrial impairment as well as apoptosis (Zhang et al. 2019). Salameh et al. also found that EGCG can protect rabbit hearts from ischemic/reperfusion (I/R) injury by increasing the ATP levels in the myocardium and reducing oxidative stress (Salameh et al. 2018). However, whether EGCG has beneficial effects on rat hearts under chronic HH conditions remains unknown.

In our experiment, the animal model of HH was made by exposing plain-grown rats to the plateau and raising them for 8 weeks. Our findings based on the model demonstrate that an 8-week duration of HH can cause injuries in the LV, especially mitochondrial damage. However, administration of EGCG significantly reversed these detrimental effects. The cardioprotective effect of EGCG may be achieved through alleviation of oxidative stress, reduction of inflammation, and the decrease in CVF, anoxic area, and apoptotic rate of cardiomyocytes. We further detected the antioxidant-related protein expression and speculated that the regulation of the endogenous antioxidant effect of EGCG is perhaps achieved in part via activating the Nrf2/HO-1-related pathway.

Consistent with previous studies, our results showed that RBC count, HGB concentration, HCT, and MCHC in the blood significantly increased, suggesting that the oxygen-carrying capacity was enhanced under chronic HH conditions. Serum levels of EPO, a glycoprotein that stimulates erythropoiesis and is secreted mainly by the kidney, also increased. These hematological adaptations allow the organs and tissues to cope better with the increased oxygen demand, while the blood viscosity may increase as a consequence (Keohane et al. 2013; Chen et al. 2022). Reportedly, excessive production of RBC and HGB can reduce blood flow velocity and exacerbate hypoxemia, which is commonly referred to as high-altitude polycythemia (HAPC) (Reeves et al. 2004), while in our experiment, the blood index of the model rats did not meet the diagnostic criteria for HAPC (HGB ≥ 210 g/L) (Liu et al. 2022). Furthermore, administration of EGCG and salidroside did not affect the alterations of the aforementioned blood indexes.

Given that high-altitude acclimation is accompanied by changes in cardiovascular properties (Chen et al. 2022; Williams et al. 2022), chronic hypoxia-induced pulmonary vasoconstriction and pulmonary hypertension may increase the afterload of the right ventricle and thereafter decrease blood return to the left heart, leading to a reduced filling condition of the LV (Williams et al. 2022). Moreover, plasma volume decreases with exposure to high altitudes. Consistent with previous studies on humans, our study found that LVEDV and LVESV decreased in the HH group while LVEF was preserved (Chen et al. 2022; Williams et al. 2022). These findings offer clues that the global cardiac function is preserved under HH conditions despite the potential for hypoxia stress to impair the contraction and relaxation of the cardiomyocytes (Allen et al. 1987). Furthermore, the LV mass was found decreased in the HH group compared to the P group, which could be explained as an adaptive change corresponding to the loss of skeletal muscle to increase oxygen utilization (Holloway et al. 2011). However, cardiac adaption to chronic HH exposure is also accompanied by myocardial injuries, as evidenced by the elevation of cardiac enzymes, including CTnI, CK, CK-MB, and LDH (Jing et al. 2020). Our experiment demonstrates that EGCG supplementation could reverse these adverse effects, indicating its cardioprotective role under chronic HH conditions.

In a chronic HH conditions, reduced oxygen availability may disrupt mitochondrial respiration, result in overproduction of ROS, and increase in oxidative stress (Gaur et al. 2021). This stress increases with elevating altitude, as demonstrated by the rising levels of lipid peroxidation (Joanny et al. 2001). Additionally, exposure to HH conditions can suppress the enzymatic antioxidant system, which may occur within a few hours after entering high altitudes. Our results find that EGCG ameliorates the ROS and MDA concentration in the experimental group along with the increase in antioxidant enzymes in the LV under chronic HH conditions. As the main organelle involved in ROS production in cells, mitochondrion is responsible for oxygen consumption and energy production, being susceptible to oxidative stress status (Xue et al. 2020). Morphologically, severely degenerated mitochondria were detected in the HH group by TEM, and a similar phenomenon has been reported to occur in the early stage of myocardial ischemia (Xue et al. 2020). As expected, EGCG treatment significantly alleviated mitochondrial injuries. These data provide evidence that EGCG may be able to protect the myocardium by regulating the endogenous antioxidant system against oxidative stress.

Since hypoxia-induced inflammation is considered to be involved in high-altitude acclimation, responses to it are regulated by an intricate interplay between molecular signaling pathways (Pham et al. 2021). Nevertheless, excessive or chronic inflammation is associated with maladjustment and disease progression (Pena et al. 2020). Under hypoxia condition, animal studies reported that the levels of inflammation in target organs are remarkably higher in the experimental group compared with normal group, which was associated with cellular injury (Jing et al. 2020; Li et al. 2020). Human research revealed that individuals who experience AMS have higher levels of circulating pro-inflammatory cytokines compared with those who do not (Wang et al. 2018). Moreover, such inflammatory responses may be related to cardiac remodeling (Pena et al. 2020). In our study, EGCG not only reduced the elevated pro-inflammatory cytokines but also inhibited the increased CVF of the LV, indicative of its potential role in inhibiting inflammation and preventing cardiac remodeling under chronic HH conditions. Despite some prior studies on the relationship between HH and inflammation, further research is still needed to determine the conditions (such as altitude, exposure time, underlying diseases) and the extent of inflammation-induced myocardial impairment.

Apoptosis is a selective cell death process that has a key role in normal growth of cardiomyocytes, but it can be involved in various pathological conditions of the heart. HH-induced myocardial injuries have been revealed to be accompanied by cellular apoptosis (Nehra et al. 2016). Bcl-2 is an essential anti-apoptotic protein that counteracts the pro-apoptotic protein Bax and inhibits the activation of caspase family proteins, which are responsible for DNA degradation as endonucleases (Lee et al. 2006). Feng et al. (2021) reveled that hypoxia could augment the concentration of BAX while decrease the Bcl-2, inducing apoptosis in cardiomyocytes. Xuan and Jian (2016) found that EGCG prevented I/R-induced cardiomyocyte death and improved heart function. In our study, TUNEL staining revealed that EGCG significantly reduced HH-induced myocardial apoptosis. Western blot further indicated that its anti-apoptotic effect could be achieved by regulating apoptosis-related proteins. However, it is noteworthy that the increased apoptotic rate of cardiomyocytes does not seem to affect the global contractile function of the LV under an 8-week HH exposure.

Nrf2 is considered to be an important modulator of redox status in cells against harmful signals and is widely expressed in oxygen-consuming organs. During stress conditions, Nrf2 is activated and translocated from the cytoplasm into the nucleus, where it binds to the antioxidant response elements and controls the downstream molecules (Taguchi et al. 2011). Among these molecules, HO-1 is one of the most important executors that protects cardiomyocytes through its antioxidant, anti-inflammation, and anti-apoptotic effects (Stefanson et al. 2014). Recently, it has been recognized that activation of the Nrf2/HO-1 pathway is beneficial in treating cardiovascular disorders including ischemic heart disease, arrhythmia, and heart failure, as well as in preventing the AMS (Lisk et al. 2013; Smith et al. 2016; Lu et al. 2020). With regard to the chronic HH environment, our experiment shows that EGCG significantly elevated the levels of Nrf2 and HO-1 in the LV, suggesting that cardioprotective property of EGCG may be achieved in part by activating this signaling pathway. However, there remain some unresolved issues that require further investigation. As reported by Sethy et al. (2011), the total level of Nrf2 increased in mouse brain exposed to acute HH conditions, which may be ascribed to the nuclear transport, whereas how Nrf2 in the LV changes during acute to chronic HH acclimation remains to be investigated. Besides, how EGCG regulates Nrf2 in cardiomyocytes needs further investigation.

## Conclusions

In summary, our study provides evidence that chronic exposure to HH environment at high altitude can cause injuries to the myocardium. EGCG supplementation exerts a cardioprotective role under such conditions, with its underlying mechanisms thought to be probably through alleviating HH-induced oxidative stress, inflammatory response, and myocardial apoptosis. It is possible that the beneficial effect of EGCG is mediated by the Nrf2 and HO-1 augmentation. Our study offers novel insights into the utilization of natural compounds to address the issues of hypoxic stress at high altitudes.
